# Isometric Hamming embeddings of weighted graphs

**DOI:** 10.1016/j.dam.2023.02.005

**Published:** 2023-02-17

**Authors:** Joseph Berleant, Kristin Sheridan, Anne Condon, Virginia Vassilevska Williams, Mark Bathe

**Affiliations:** a Department of Biological Engineering, Massachusetts Institute of Technology, Cambridge, MA, United States of America; b Department of Computer Science, University of Texas at Austin, United States of America; c Department of Computer Science, University of British Columbia, Vancouver, Canada; d Computer Science and Artificial Intelligence Laboratory, Massachusetts Institute of Technology, Cambridge, MA, United States of America

**Keywords:** Isometric embeddings, Graph embeddings, Metric spaces, Weighted graphs, Hamming graphs, Graph factorization

## Abstract

A mapping α:V(G)→V(H) from the vertex set of one graph G to another graph H is an *isometric embedding* if the shortest path distance between any two vertices in G equals the distance between their images in H. Here, we consider isometric embeddings of a weighted graph G into unweighted Hamming graphs, called Hamming embeddings, when G satisfies the property that every edge is a shortest path between its endpoints. Using a Cartesian product decomposition of G called its *canonical isometric representation*, we show that every Hamming embedding of G may be partitioned into a *canonical partition*, whose parts provide Hamming embeddings for each factor of the canonical isometric representation of G. This implies that G permits a Hamming embedding if and only if each factor of its canonical isometric representation is Hamming embeddable. This result extends prior work on unweighted graphs that showed that an unweighted graph permits a Hamming embedding if and only if each factor is a complete graph. When a graph G has nontrivial isometric representation, determining whether G has a Hamming embedding can be simplified to checking embeddability of two or more smaller graphs.

## 1. Introduction

*Isometric embeddings*, or distance-preserving mappings from the vertices of one graph to another, are well studied for unweighted graphs but remain relatively unstudied for weighted graphs. Such embeddings are useful whenever a graph’s distance metric is of primary interest, and representation in another graph can simplify analysis or manipulation of graph distances. Of particular interest are embeddings into Hamming graphs, or products of complete graphs. For example, Hamming graphs may be used in molecular engineering to represent DNA sequence variants (e.g., [12]) so that embedding a graph aids the design of DNA strands with variable sequence similarity, for applications requiring DNA with non-orthogonal pairwise binding interactions [1,4]; in communication theory to permit maximally efficient information routing without inspecting the global network structure [16]; in linguistics to relate the closeness of linguistic objects to simpler predicate vector models [15]; and in coding theory to optimize error-checking codes based on Hamming distance [18].

**Fig. 1.**
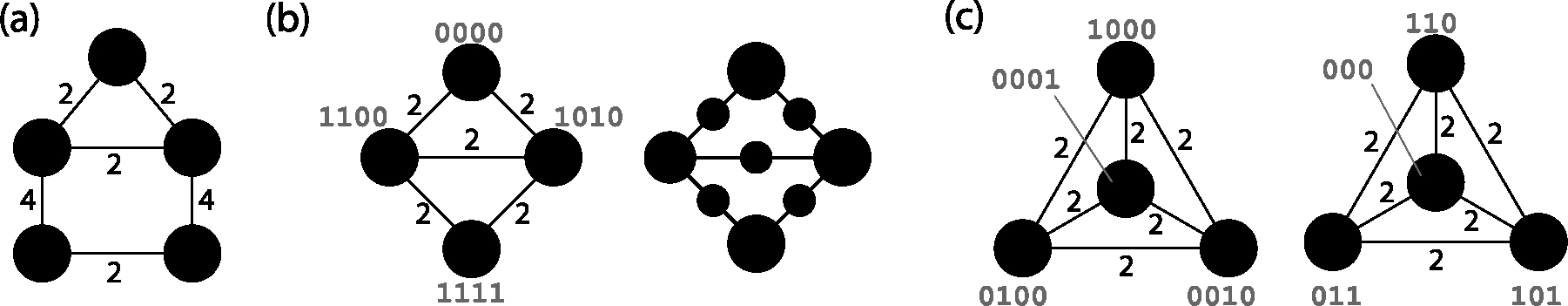
Embedding weighted graphs is more difficult than unweighted graphs, and some guarantees for unweighted graphs no longer hold. (a) A simple graph which permits a Hamming embedding into ${\left(K_{2}\right)}^{4}\times {\left(K_{3}\right)}^{2}$, but which is not covered by previous theorems on isometric embeddings of weighted graphs. (b) A weighted graph that permits a hypercube embedding, but for which the unweighted graph generated via edge subdivision is not. (c) A weighted graph, $K_{4}$ with uniform edge weights of 2, for which multiple non-equivalent hypercube embeddings exist.

Finding isometric embeddings into a particular destination graph or determining their existence is nontrivial, even with simple graphs like Hamming graphs. A large body of work addresses isometric embeddings of unweighted graphs [2,11,14,17,23,24] but studies of weighted graphs have considered only embeddings of limited classes of graphs into hypercubes [8,20,22]. Our interest in the isometric graph embedding problem initially stemmed from molecular engineering, in which the metrics we wish to embed are generally complex and rarely fall into a previously studied graph type (Fig. 1a). Naïve attempts to convert weighted graphs into unweighted graphs via edge subdivision can produce unweighted graphs without an isometric embedding when the original weighted graph did permit such an embedding (Fig. 1b). In addition, weighted graphs may have multiple non-equivalent isometric embeddings into a single destination graph (Fig. 1c), which does not occur with unweighted graphs [17]. Indeed, the concept of isometric representation in Cartesian graph products, which is central to the study of isometric embeddings for unweighted graphs, has only recently been extended to weighted graphs [21].

The results of this paper apply specifically to those weighted graphs for which each edge is a shortest path between its endpoints, which we call *weight-minimal* graphs.^2^ Such graphs are natural to study in the context of isometric embeddings because an edge with weight greater than the distance between its endpoints will not affect the graph’s shortest path metric. All our results apply also to *edge-minimal* graphs, which are those graphs for which each edge is the unique shortest path between its endpoints.

When only the distances in a graph are of interest, our results apply to any graph with positive edge weights, because the graph can be made weight- or edge-minimal through edge removal, without affecting distances. Similarly, our results apply to any finite metric space by representing it in a weight- or edge-minimal graph. Notably, multiple graphical realizations generally exist for a given finite metric space [9], and each may have different embedding properties.

### 1.1. Prior work

#### 1.1.1. Isometric representation of graphs in Cartesian products

**Fig. 2.**
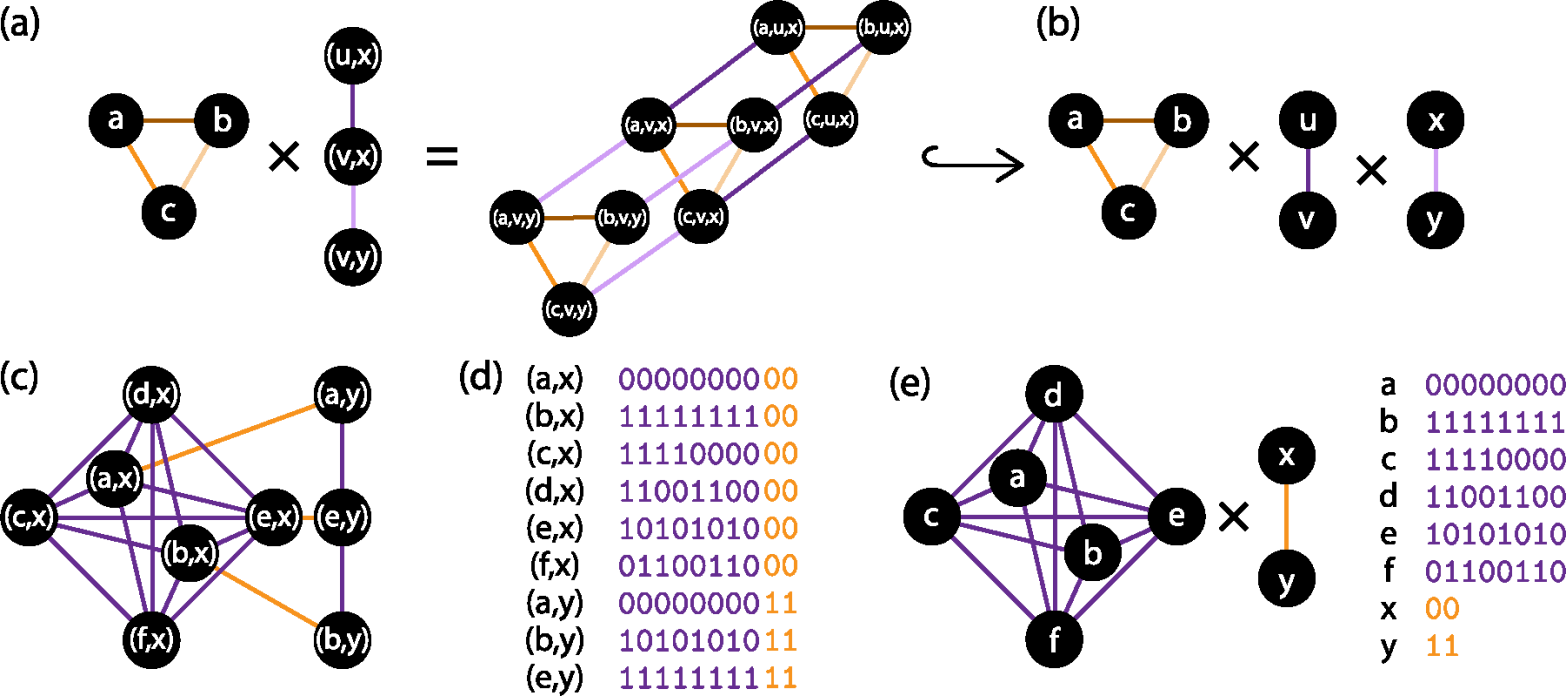
(a) Illustration of the Cartesian graph product. Edge colors indicate correspondence between edges in the factor and product graphs. If edges are weighted, two edges of the same color will have the same weight. (b) This graph product is isometrically embeddable in a product of three irreducible graphs ($K_{3},\;K_{2}$, and $K_{2}$), which form the factors of its canonical isometric representation. (c) A weighted graph, with purple edges of weight 4 and orange edges of weight 2. Edge colors also indicate equivalence classes under the transitive closure $\overset{ˆ}{\theta }$ of the Djoković-Winkler relation (d) A hypercube embedding of this graph, with digits colored to indicate equivalence classes under $\overset{ˆ}{\gamma }.\;\overset{ˆ}{\gamma }$ is the transitive closure of γ, which relates coordinates whose digits change together across some edge. (e) Our results show that the hypercube embedding in (d) may be partitioned into a hypercube embedding for each factor. The same is true of any Hamming embedding of a weight-minimal graph.

The *Cartesian graph product* of *k* ≥ 1 graphs is a graph whose vertex set is the Cartesian product of the vertex sets of each factor graph, and whose edge set is such that each edge of the product graph corresponds to a single edge from a single one of the factors (Fig. 2a). Cartesian graph products have the important property that every distance in the product graph can be decomposed additively into distances in the factor graphs. This property makes Cartesian products relevant to isometric embeddings, because the product graph’s shortest path distance metric is represented in the distance metrics of the factor graphs. An *isometric representation* of G is an isometric embedding of G into a Cartesian product of graphs with additional conditions to avoid unnecessary vertices and edges.^3^ An *irreducible* graph is one whose isometric representations always include itself as a factor. In their work on unweighted graphs, Graham and Winkler [17] found that every connected, unweighted graph has a unique *canonical isometric representation* in a product of irreducible graphs (Fig. 2b).

Sheridan et al. [21] generalized the notion of isometric representation to weight-minimal weighted graphs. In close analogy to unweighted graphs, they showed that any connected weight-minimal graph has a unique canonical isometric representation in a product of irreducible weight-minimal graphs, and that the canonical isometric representation can be found in polynomial time. To do so, they made use of the Djoković-Winkler relation θ on the edge set of a graph, and its transitive closure $\overset{ˆ}{\theta }$ (Fig. 2c). In particular, they showed that, given any weight-minimal graph, there is a bijection between its equivalence classes under $\overset{ˆ}{\theta }$ and the factors of its canonical isometric representation.

#### 1.1.2. Hypercube and Hamming embeddings of unweighted graphs

A unique canonical isometric representation exists for all unweighted or weighted graphs. However, additional structural results apply when an unweighted graph is an isometric subgraph of a hypercube or a Hamming graph. In the former case, the graph is called a *partial cube*. Throughout the paper, all hypercube and Hamming graphs are unweighted.

Hypercube embeddings were considered in 1965 by Firsov [15], who provided preliminary results on the existence of graphs without such embeddings. The first full characterization of unweighted partial cubes was discovered by Djoković [11]. For an edge uv of an unweighted graph G, Djoković defined G(u,v) as the subgraph induced by all vertices closer to u than v, and showed G is a partial cube if and only if G is bipartite and, for every edge uv,G(u,v) and G(v,u) are convex (i.e., closed under the inclusion of shortest paths). Winkler [24] extended these results to show that the hypercube embedding of any unweighted graph is unique, up to symmetries of the hypercube, and also provided an alternate characterization of unweighted partial cubes as those unweighted bipartite graphs G for which the Djoković-Winkler relation θ is transitive. Several particular classes of graphs are known to be partial cubes, including trees, median graphs (which are in fact the retracts of hypercubes) [3], benzenoid graphs [19], and tope graphs of oriented matroids [5].

Several of these results generalize to isometric subgraphs of Hamming graphs. Winkler [24] proved that, as with hypercube embeddings, the isometric embedding of an unweighted G into a Hamming graph is unique, up to symmetries of the Hamming graph. He also proved that an unweighted graph G is an isometric subgraph of a power of $K_{3}$ if and only if θ is transitive. Later, Chepoi [6] gave a complete characterization of isometric subgraphs of Hamming graphs as exactly those unweighted graphs G for which $G\left(u,v\right),G(v,u),G_{=}(u,v)$, and their complements in G are all convex for every edge uv, where $G_{=}(u,v)$ is the subgraph induced by the set of vertices equidistant to u and v. Wilkeit [23] provided a novel polynomial-time algorithm for computing Hamming embeddings of unweighted G, and used this algorithm to prove additional properties of unweighted isometric subgraphs of Hamming graphs, such as satisfying the pentagonal inequalities.

Shpectorov [22] characterized the unweighted graphs that permit scale embeddings into hypercubes (i.e., embeddings into hypercubes for which the distances are scaled by a constant factor), which are exactly those graphs with $\ell_{1}$-embeddings, called $\ell_{1}$-graphs. He showed that any $\ell_{1}$-graph is an isometric subgraph of a Cartesian product of unweighted cocktail party graphs^4^ and unweighted halved cubes. Further, if unweighted complete graphs are allowed in this product, then there is a product with the property that the “columns” of any scale hypercube embedding may be partitioned so that each part is a scale embedding of a factor in the Cartesian product.

For unweighted graphs, hypercube and Hamming embeddability can be tested in polynomial time. The fastest known algorithm for testing hypercube embeddability was provided by Eppstein in 2008 [13], and works in $O\left(n^{2}\right)$ time. Hamming embeddings may be computed in $O\left(n^{3}\right)$ time [23]. For scale embeddings, Deza and Shpectorov published an O(nm)-time algorithm for testing $\ell_{1}$-embeddability [10]. These polynomial-time results do not apply in general for weighted graphs.

#### 1.1.3. Hypercube embeddings of weighted graphs

Weighted graphs may represent a larger class of metric spaces than unweighted graphs, and isometric embeddings of weighted graphs are correspondingly more difficult. Work on finding isometric embeddings of weighted graphs has been confined to embeddings into hypercubes. In contrast to the unweighted case, determining if an arbitrary weighted graph permits a hypercube embedding is NP-hard [7].

However, polynomial-time methods for determining hypercube embeddability are known for particular classes of weighted graphs. For example, weighted graphs that may be tested for hypercube embeddability in polynomial time include line graphs and cycle graphs [8], as well as graphs whose distances come from the set {x,y,x+y} for integers x,y at least one of which is odd [20].

Shpectorov’s work on scale embeddings of unweighted graphs also has an interpretation in terms of weighted hypercube-embeddable graphs with uniform edge weights, namely, that such a graph must have an isometric representation in a Cartesian product of cocktail party graphs, half-cube graphs, and complete graphs. This follows from the fact that the canonical isometric representation of a graph with uniform edge weights k is identical to that of the corresponding unweighted graph, with the addition of edge weights of k in each factor. This means that the O(nm) algorithm for testing $\ell_{1}$-embeddability applies to uniformly weighted graphs also.

As mentioned previously, any scale hypercube embedding may be partitioned to provide hypercube embeddings of each factor in the canonical isometric representation. Our results generalize this result of Shpectorov from weighted graphs with uniform edge weights to those with arbitrary positive weights.

With weighted graphs the isometric embedding of a graph may no longer be unique, as noted previously (e.g., Fig. 1c). When multiple isometric embeddings exist, some results on counting the number of such embeddings have been established; for example, Deza and Laurent showed that $K_{4}$ with uniform edge weights of integer 2k always has k+1 unique isometric embeddings into hypercubes [9].

### 1.2. Our results

In this work, we develop a formal relationship between isometric representations of weight-minimal weighted graphs and their isometric embeddings into unweighted Hamming graphs. Our results may be extended in full to isometric embeddings into hypercubes, and in part to isometric $\ell_{1}$-embeddings. One of the two main results of this paper is the following theorem, which states that a weight-minimal graph G permits a Hamming embedding if and only if each factor of its canonical isometric representation permits a Hamming embedding. This result may be contrasted with the unweighted case [17], in which each factor must *be* a complete graph.

#### Theorem 1.1.

A weight-minimal weighted graph G has a Hamming embedding if and only if a Hamming embedding exists for each factor of its canonical isometric representation. Similarly, G has a hypercube embedding or $\ell_{1}$-embedding if and only if each factor of its canonical isometric representation has a hypercube embedding or $\ell_{1}$-embedding, respectively.

Theorem 1.1 is proven by Theorem 3.5 and Corollaries 3.6 and 3.7 in Section 3. We briefly sketch the proof and the novel contributions necessary for it here. A Hamming embedding of weighted graph $G=\left(V(G),E(G),w_{G}\right)$ can be written as a mapping $\eta \;:V(G)\to \Sigma^{m}$, where m is the embedding *dimension*, Σ is the embedding alphabet, and the distance between two elements of $\Sigma^{m}$ is given by Hamming distance. η may be partitioned, that is, the columns may be grouped to form embeddings of lower dimension (though these are not generally isometric). The second main result of this manuscript is a proof of existence for a special *canonical partition* of η that provides Hamming embeddings for each factor of the canonical isometric representation of G.

#### Theorem 1.2.

Let $G=\left(V\left(G\right),E(G),w_{G}\right.)$ be a weight-minimal weighted graph and η a Hamming (hypercube) embedding of G. Let the factors of the canonical isometric representation of G be the graphs $G_{1},\ldots ,G_{n}$. Then there exists a partition of η into embeddings $\eta^{1},\ldots ,\eta^{n}$ such that $\eta^{i}$ forms a Hamming (hypercube) embedding of $G_{i}$.

Theorem 1.2 is proven by Propositions 3.3 and 3.4. To prove Theorem 1.1, we use Theorem 1.2 to show that if G is Hamming embeddable then the factors of its canonical representation are also Hamming embeddable. The converse is easily shown to be true, by concatenating Hamming embeddings of each factor. To construct the canonical partition of η, we introduce a novel relation, called γ, on the coordinates of $\eta =\left(\eta_{1},\ldots ,\eta_{m}\right)$. Informally, two coordinates are related by γ if the corresponding digits of η change across some edge in E(G). The transitive closure of γ is an equivalence relation $\overset{ˆ}{\gamma }$, and its equivalence classes define a partition of the digits of η, which is the canonical partition of η (Fig. 2c–d). Much of our effort is spent proving the existence of a bijection between the equivalence classes under $\overset{ˆ}{\theta }$ (i.e. sets of edges) and the equivalence classes under $\overset{ˆ}{\gamma }$ (i.e. sets of coordinates), which we use to construct a bijection between the factors of the canonical isometric representation of G and the canonical partition of any Hamming embedding of G. As a final step, we prove that the embeddings of this partition form Hamming embeddings of each factor (Fig. 2e).

Using Theorem 1.2, we are also able to prove the following result on the number of non-equivalent Hamming embeddings or hypercube embeddings of G. We define equivalence of two Hamming embeddings in the same way as Winkler in [24]; the formal definition is given in Section 2. Informally, two Hamming embeddings are equivalent if they can be made identical by permuting coordinates or coordinate values (i.e., swapping the positions of some number of $\eta_{i}$ and/or substituting the values of particular $\eta_{i}$). The following theorem is proven by Corollary 3.8.

#### Theorem 1.3.

Given a weight-minimal weighted graph G, the number of non-equivalent Hamming embeddings of G is the product of the number of non-equivalent Hamming embeddings of each factor of its canonical isometric representation. Similarly, the number of non-equivalent hypercube embeddings of G is the product of the number of non-equivalent hypercube embeddings of each factor of its canonical isometric representation.

These theorems imply an important structure of any Hamming embedding of a graph G: such an embedding must be equivalent to a concatenation of Hamming embeddings for each factor of the canonical isometric representation of G. As a consequence, the existence of a Hamming embedding of G implies one for each factor. The converse is easily shown to be true also. In practice, these results mean that we may recognize graphs that do not permit a Hamming embedding by analyzing the factors of a canonical isometric representation, which may be significantly smaller. They also allow us to extend polynomial-time results for determining Hamming or hypercube embeddability of a graph to graphs whose canonical isometric representation factors can be determined as Hamming or hypercube embeddable in polynomial time. These include Cartesian products of line graphs, cycle graphs, graphs with distances in {x,y,x+y} for integers x,y at least one odd, and graphs with uniform edge weights [8,20,22], along with any other graphs later found to be Hamming or hypercube embeddable. Significant additional work remains in characterizing classes of irreducible weighted graphs for which Hamming or hypercube embeddings may be constructed efficiently. However, our work significantly eases the problem of finding Hamming or hypercube embeddings for graphs with nontrivial isometric representations, and is also a step forward in better understanding isometric embeddings of weighted graphs into more complex destination graphs.

## 2. Preliminaries

In this paper, all graphs are finite, connected, and undirected. Except where specified, all graphs are weighted with the exception of Hamming graphs (defined below) which are always unweighted. We use $V\left(G\right),\;E(G)$, and $w_{G}:E(G)\to Z_{>0}$ to denote the vertex set, edge set, and weight function of a graph G, respectively. An edge between vertices u,v∈V(G) is written uv or vu; since all edges are undirected, uv∈E(G) implies vu∈E(G). The distance from u to v,u,v∈V(G), is the minimum edge weight sum along a path from u to v, denoted $d_{G}\;:V(G)\times V(G)\to Z_{\geq 0}$.

The following defines two types of graphs that are minimal with respect to a distance metric. Existence and uniqueness of canonical isometric representation has been proven for weighted graphs in these classes [21].

### Definition 2.1.

A graph G is **weight-minimal** if every edge in E(G) forms a shortest path between its endpoints. That is, $w_{G}(uv)=d_{G}(u,v)$ for all uv∈E(G). If every edge forms a unique shortest path between its endpoints then G is **edge-minimal.**

In this manuscript, we assume all graphs are weight-minimal. Our results apply equally to edge-minimal graphs, as edge-minimality implies weight-minimality. Note that any unweighted graph is edge-minimal and that any weighted graph may be made edge-minimal by removing any edges not satisfying the condition in Definition 2.1. In addition, every finite metric space can be realized as an edge-minimal graph to which our results may be applied.

The Cartesian graph product of one or more graphs $G_{1},\ldots ,G_{n}$ is written $G=G_{1}\times \cdots \times G_{n}$ or $G=\prod_{i=1}^{n}G_{i}.\;G$ is defined with $V(G)=V\left(G_{1}\right)\times \cdots \times V\left(G_{n}\right)$, two vertices $\left(u_{1},\ldots ,u_{n}\right)$ and $\left(v_{1},\ldots ,v_{n}\right)$ adjacent if and only if there is exactly one j such that $u_{j}v_{j}\in E\left(G_{j}\right)$ and $u_{i}=v_{i}$ for all i≠j, and $w_{G}(uv)=w_{G_{j}}\left(u_{j}v_{j}\right)$ for j chosen as above (Fig. 2a). The Cartesian graph product has the following important distance property:

(1) $$d_{G}\left(u,v\right)=\sum_{i=1}^{n}d_{G_{i}}\left(u_{i},v_{i}\right)$$

where $u=\left(u_{1},\ldots ,u_{n}\right)$ and $v=\left(v_{1},\ldots ,v_{n}\right)$. This implies that any path in G can be decomposed into a set of paths in the $G_{i}$, with the path length in G equal to the sum of the path lengths in the $G_{i}$.

A graph embedding $\pi \;:V(G)\to V(G^{*})$ of a graph G into a graph $G^{*}$ maps vertices of G to those of $G^{*}$. If π satisfies $d_{G}(u,v)=d_{G^{*}}(\pi (u),\;\pi (v))$ for all u,v∈V(G), then π is an isometric embedding. When such a π exists, we say that $G↪G^{*}$. For convenience, we let $d_{\pi }(u,v)=d_{G^{*}}(\pi (u),\pi (v))$.

When G has an isometric embedding into a Cartesian graph product (e.g., Fig. 2b) and also satisfies certain redundancy constraints, we call this embedding an isometric representation. The following definition is due to Sheridan et al. [21]:

### Definition 2.2.

Consider graphs G and $G^{*}=\prod_{i=1}^{n}G_{i}^{*}$. If an embedding $\pi \;:V(G)\to V(G^{*}),\;\pi =\left(\pi_{1},\ldots ,\pi_{n}\right)$, exists satisfying the following criteria:

1. $d_{G}\left(u,u^{'}\right)=d_{G^{*}}\left(\pi (u),\pi \left(u^{'}\right)\right)$,
2. uv∈E(G) implies $\pi (u)\pi (v)\in E(G^{*})$ and $w_{G}(uv)=w_{G^{*}}(\pi (u)\pi (v))$,
3. every vertex in $G_{i}^{*}$ is in the image of $\pi_{i},\;1\leq i\leq n$, and
4. every edge $u_{i}v_{i}$ in $G_{i}^{*}$ equals $\pi_{i}(u)\pi_{i}(v)$ for some uv∈E(G)

then we say that π is an **isometric representation** of G and refer to each $G_{i}^{*}$ as a factor of π.

A graph G is *irreducible* if all its isometric representations include itself as a factor. An isometric representation in irreducible factor graphs is called an irreducible isometric representation. For convenience, we assume that no factor of an isometric representation equals $K_{1}$, except when $G=K_{1}$.

Informally, Definition 2.2 requires both that G be isometrically embeddable into $G^{*}$ and that edges be preserved within this embedding. This second condition is a natural one for manipulating graph structures. The final two conditions ensure that there are no unnecessary vertices and edges in the factors, i.e., the representation is irredundant [17].

Sheridan et al. [21] showed that an irreducible isometric representation of a weighted graph is unique. This isometric representation is called its *canonical isometric representation* or *canonical isometric embedding*. The authors used the Djoković-Winkler relation θ, which we restate here as has been defined elsewhere [17,21]. For a graph G, two edges in the graph uv,ab∈E(G) are related by θ if and only if:

(2) $$\left[d_{G}(u,a)-d_{G}(u,b)\right]-\left[d_{G}\left(v,a\right)-d_{G}\left(v,b\right)\right]\neq 0.$$

We note that θ is symmetric and reflexive. Let the equivalence relation $\overset{ˆ}{\theta }$ be the transitive closure of θ.

Algorithm 1 of Sheridan et al. [21] constructs the canonical isometric representation of a weighted graph G similarly to the corresponding construction for unweighted graphs described by Graham and Winkler [17], with a slight modification to handle the addition of edge weights. We describe this algorithm, which will be used in Section 3 to prove an important property of the canonical isometric representation of weighted graphs. Let $E(G)/\overset{ˆ}{\theta }=\left\{E_{1},\ldots ,E_{n}\right\}$ be the set of equivalence classes of $\overset{ˆ}{\theta }$. Then, for each $E_{i}$, let $G_{i}$ be the graph with vertices taken as the connected components of $G\backslash E_{i}$. Two vertices form an edge $e^{*}$ if there is an edge $e\in E_{i}$ between the corresponding connected components, with $w_{G_{i}}(e^{*})=w_{G}(e)$. The $G_{i}$ formed by these steps are exactly the factors of the canonical isometric representation of G.

Finally, we introduce our notation for Hamming graphs. A Hamming graph is a product of complete graphs, and in this paper will always be unweighted. For our purposes, it will be most convenient to represent a Hamming graph as a graph on all strings of a fixed length, with edges between strings that differ at a single position. Then Hamming graph H has $V(H)=\prod_{i=1}^{m}\;\Sigma_{i}$, where $\Sigma_{i}$ is the alphabet for the ith coordinate and m the dimension of H. Distance in H equals the Hamming distance between pairs of vertices. H is a hypercube graph if $\left|\Sigma_{i}\right|=2$ for each i,1≤i≤m. For Hamming or hypercube graph H, an isometric embedding η:V(G)→V(H) is a Hamming or hypercube embedding, respectively. We use subscripts to refer to individual letters in the image of $\eta =\left(\eta_{1},\ldots ,\eta_{m}\right)$ or, equivalently, $\eta =\eta_{1}\cdots \eta_{m}$. The indices [m] of η are its coordinates, where [m] denotes the first m positive integers, and each $\eta_{i},\;1\leq i\leq m$, is a digit. Two or more embeddings $\eta^{1},\ldots ,\eta^{n}$ of dimensions $m_{1},\ldots ,m_{n}$ may be concatenated, forming an embedding $\eta =\eta^{1}\cdots \eta^{n}=\eta_{1}^{1}\cdots \eta_{m_{1}}^{1}\cdots \eta_{1}^{n}\cdots \eta_{m_{n}}^{n}$.

Two Hamming embeddings $\eta ,\;\eta^{\prime }$ are equivalent if there exists a permutation σ of the m coordinates and m bijections $\beta_{i},\;1\leq i\leq m$, such that $\eta^{\prime }(u)=\beta_{1}(\eta_{\sigma (1)}(u))\cdots \beta_{m}(\eta_{\sigma (m)}(u))$ for all u∈V(G). A Hamming embedding of G is unique if it is equivalent to all other Hamming embeddings of G. We assume there are no unnecessary digits in η (i.e., every digit changes across some edge), which is analogous to our assumption that $K_{1}$ is not a factor of any isometric representation.

For any two vertices u,v∈V(G), we define the function $D_{\eta }$ as follows:

(3) $$D_{\eta }\left(u,v\right)=\left\{j\in \left[m\right]\;:\eta_{j}\left(u\right)\neq \eta_{j}\left(v\right)\right\},$$

noting that a graph G with Hamming embedding η:V(G)→V(H) will have $d_{G}(u,v)=d_{\eta }(u,v)=\left|D_{\eta }(u,v)\right|$. When uv is an edge, we say that the digits indicated by $D_{\eta }(u,v)$ change across uv. We also introduce a relation γ, which relates two coordinates of an embedding if both corresponding digits change across any edge:

(4) $$j\gamma j^{\prime }⟺\exists uv\in E\left(G\right),\left\{j,j^{\prime }\right\}\subseteq D_{\eta }\left(u,v\right).$$

Let $\overset{ˆ}{\gamma }$ be the transitive closure of γ. Since γ is symmetric and reflexive, $\overset{ˆ}{\gamma }$ is an equivalence relation.

A *partition* $\left\{\eta^{1},\ldots ,\eta^{n}\right\}$ of an m-dimensional Hamming embedding η is defined by a partition of its coordinates [$m],\;\left\{J_{1},\ldots ,J_{n}\right\}$, with each $\eta^{i}$ equal to the projection of η onto the coordinates in $J_{i}$. Let $[m]/\overset{ˆ}{\gamma }$ be the set of equivalence classes of [m] under $\overset{ˆ}{\gamma }$. Then $[m]/\overset{ˆ}{\gamma }$ defines a partition of η, which we call its *canonical partition*. This terminology is motivated by Proposition 3.3, which guarantees a bijection between the canonical partition of η and the factors of the canonical isometric representation of G.

## 3. Structure of Hamming embeddings of weighted graphs

In this section, we construct a bijection between the canonical partition of any Hamming embedding of a graph G and the factors of its canonical isometric representation. This result is used to prove the two main findings of the paper: Proposition 3.4, which proves that the canonical partition of a Hamming embedding of G forms a Hamming embedding for each factor of its canonical isometric representation; and Theorem 3.5, which proves that G permits a Hamming embedding if and only if each factor of its canonical isometric representation also permits a Hamming embedding.

We begin with the following lemma, which will be useful in this section and is also a useful observation about the canonical isometric representation of weighted graphs.

### Lemma 3.1.

Consider the canonical isometric representation of $G,\;\pi \;:V(G)\to V\left(\prod_{i=1}^{n}G_{i}\right)$, as constructed by Algorithm 1 of Sheridan et al. [21], and assume each $G_{i}$ was constructed from equivalence class $E_{i}\in E(G)/\overset{ˆ}{\theta }$. Let $\pi =\left(\pi_{1},\ldots ,\pi_{n}\right)$. For u,v∈V(G) and a path P from u to v, let $P_{i}$ be the subsequence of edges in P that are in $E_{i}$. Then there is a path in $G_{i}$ from $\pi_{i}(u)$ to $\pi_{i}(v)$ of length equal to the sum of the edge weights in $P_{i}$.

### Proof.

By inspection of Algorithm 1 (see Section 2), the endpoints of any edge $xy\in E(G)\backslash E_{i}$ will be mapped to the same node in $G_{i}$ (i.e., $\pi_{i}(x)=\pi_{i}(y)$). Let $ab\in P_{i}$. Since π is an isometric embedding of $G,\;w_{G}(ab)=d_{G}(a,b)=\sum_{j=1}^{n}d_{\pi_{j}}(a,b)=d_{\pi_{i}}(a,b)$, where the final equality is due to the fact that a summand is zero when i≠j. As a result, there is a path from $\pi_{i}(a)$ to $\pi_{i}(b)$ of length $w_{G}(ab)$. Any edges in P but not $P_{i}$ do not change the value of $\pi_{i}$. So adjacent edges in $P_{i}$ share an endpoint, and there is a path in $G_{i}$ of length equal to the sum of the edge weights in $P_{i}$.

### Lemma 3.2.

Let G be a weighted graph with some Hamming embedding η. Let $uv,\;u^{\prime }v^{\prime }\in E(G)$, $j\in D_{\eta }(u,v)$, and $j^{\prime }\in D_{\eta }\left(u^{\prime },v^{\prime }\right)$. Then we have

$$uv\overset{ˆ}{\theta }u^{\prime }v^{\prime }⟺j\overset{ˆ}{\gamma }j^{\prime }.$$

### Proof.

To see that $uv\overset{ˆ}{\theta }u^{\prime }v^{\prime }$ implies $j\overset{ˆ}{\gamma }j^{\prime }$, observe that jγ^j′ implies that $D_{\eta }(u,v)$ and $D_{\eta }\left(u^{\prime },v^{\prime }\right)$ are disjoint. Thus,

$$\begin{array}{ll} & \left[d_{G}\left(u,u^{\prime }\right)-d_{G}\left(u,v^{\prime }\right)\right]-\left[d_{G}\left(v,u^{\prime }\right)-d_{G}\left(v,v^{\prime }\right)\right]\\ & \;=\sum_{s\in [m]}\left[d_{\eta_{s}}\left(u,u^{\prime }\right)-d_{\eta_{s}}\left(u,v^{\prime }\right)\right]-\left[d_{\eta_{s}}\left(v,u^{\prime }\right)-d_{\eta_{s}}\left(v,v^{\prime }\right)\right]=0 \end{array}$$

because each summand is nonzero only if both $\eta_{s}(u)\neq \eta_{s}(v)$ and $\eta_{s}\left(u^{\prime }\right)\neq \eta_{s}\left(v^{\prime }\right)$. So jγ^j′ implies uvθu′v′, and $uv\theta u^{\prime }v^{\prime }$ implies $j\overset{ˆ}{\gamma }j^{\prime }$. If instead $uv\overset{ˆ}{\theta }u^{\prime }v^{\prime }$ then some sequence of edges ${\left(e_{i}\right)}_{i=1}^{l}$ satisfies $uv\;\theta \;e_{1}\;\theta \cdots \theta \;e_{l}\;\theta u^{\prime }v^{\prime }$, and the transitivity of $\overset{ˆ}{\gamma }$ implies $j\overset{ˆ}{\gamma }j^{\prime }$.

To prove that $j\overset{ˆ}{\gamma }j^{\prime }$ implies $uv\overset{ˆ}{\theta }u^{\prime }v^{\prime }$, let $S=D_{\eta }(u,v)\cap D_{\eta }\left(u^{\prime },v^{\prime }\right)$, the set of all coordinates that change across both uv and $u^{\prime }v^{\prime }$. We distinguish two cases:

**Case 1** (|S|>0) : Assume |S|>0. Consider any path P with l edges, beginning with uv and ending with $u^{\prime }v^{\prime },\;l\geq 2$. We show by induction on l that $uv\overset{ˆ}{\theta }u^{\prime }v^{\prime }$. As a base case, consider l=2 and without loss of generality let $P=\left(u,v=u^{\prime },v^{\prime }\right)$. As above, we consider

$$\begin{array}{ll} & \left[d_{\eta }\left(u,u^{\prime }\right)-d_{\eta }\left(u,v^{\prime }\right)\right]-\left[d_{\eta }\left(v,u^{\prime }\right)-d_{\eta }\left(v,v^{\prime }\right)\right]\\ & \;=\sum_{s\in S}\left[d_{\eta_{s}}\left(u,u^{\prime }\right)-d_{\eta_{s}}\left(u,v^{\prime }\right)\right]-\left[d_{\eta_{s}}\left(v,u^{\prime }\right)-d_{\eta_{s}}\left(v,v^{\prime }\right)\right] \end{array}$$

with the sum restricted to coordinates s∈S for which the summand may be nonzero. Because $v=u^{\prime },\;\eta_{s}(v)=\eta_{s}\left(u^{\prime }\right)$, so $d_{\eta_{s}}\left(v,u^{\prime }\right)=0$ and $d_{\eta_{s}}\left(u,u^{\prime }\right)=d_{\eta_{s}}\left(v,v^{\prime }\right)=1$. Thus, each summand is at least +1 and the summation is at least +|S|, so $uv\;\theta \;u^{\prime }\;v^{\prime }$. Now consider the case l>2 and $P=\left(u_{0}=u,u_{1}=v,\ldots ,u_{l-1}=u^{\prime },u_{l}=v^{\prime }\right)$. If every edge $u_{i-1}u_{i},\;1<i<l$, has S and $D_{\eta }\left(u_{i-1},u_{i}\right)$ disjoint, then $\eta_{s}(v)=\eta_{s}\left(u^{\prime }\right)$ for all s∈S and as in the base case we have $uv\;\theta \;u^{\prime }\;v^{\prime }$. If some edge $u_{i-1}u_{i}$ has S and $D_{\eta }\left(u_{i-1},u_{i}\right)$ not disjoint, then consider the subpaths $P_{1}=\left(u_{0},u_{1},\ldots ,u_{i}\right)$ and $P_{2}=\left(u_{i-1},u_{i},\ldots ,u_{l}\right)$, with $l_{1}$ and $l_{2}$ edges, respectively. Clearly $2\leq l_{1},\;l_{2}<l$, so by induction we conclude that $uv\overset{ˆ}{\theta }u_{i-1}u_{i}\overset{ˆ}{\theta }u^{\prime }v^{\prime }$.

**Case 2** (|S|=0) : We are given $j\overset{ˆ}{\gamma }j^{\prime }$, so construct a sequence of coordinates $j_{0}=j,\ldots ,j_{l}=j^{\prime }$ such that $j_{i-1}\gamma j_{i}$ for all 1≤i≤l. For each pair of coordinates $j_{i-1},j_{i}$ there is some edge $e_{i}$ across which both $\eta_{j_{i-1}}$ and $\eta_{j_{i}}$ change. By Case 1, we have $uv\overset{ˆ}{\theta }e_{1},\;e_{l}\overset{ˆ}{\theta }u^{\prime }v^{\prime }$, and $e_{i-1}\overset{ˆ}{\theta }e_{i}$ for 1<i≤l. Thus, $uv\overset{ˆ}{\theta }u^{\prime }v^{\prime }$ as desired.

### Proposition 3.3.

Let G be a weighted graph with $G_{1},\ldots ,G_{n}$ the factors of its canonical isometric representation and η an m-dimensional Hamming embedding of G. Then there is a bijection between $E(G)/\overset{ˆ}{\theta }$ and $[m]/\overset{ˆ}{\gamma }$. It follows that there is a natural bijection from the set $\left\{G_{1},\ldots ,G_{n}\right\}$ to the canonical partition of η.

### Proof.

We first construct a bijection from $E(G)/\overset{ˆ}{\theta }$ to $[m]/\overset{ˆ}{\gamma }$. Let $[uv{]}_{\overset{ˆ}{\theta }}\in E(G)/\overset{ˆ}{\theta }$ contain uv∈E(G) and $[j{]}_{\overset{ˆ}{\gamma }}\in [m]/\overset{ˆ}{\gamma }$ contain j∈[m]. Then we consider the mapping $f_{2}\;:[uv{]}_{\overset{ˆ}{\theta }}\mapsto [j{]}_{\overset{ˆ}{\gamma }}$ where $j\in D_{\eta }(u,v)$. By Lemma 3.2, $f_{2}$ is injective because for $j\in D_{\eta }(u,v)$ and $j^{\prime }\in D_{\eta }\left(u^{\prime },v^{\prime }\right),\;f_{2}\left([uv{]}_{\overset{ˆ}{\theta }}\right)=f_{2}\left({\left[u^{\prime }v^{\prime }\right]}_{\overset{ˆ}{\theta }}\right)$ implies $[j{]}_{\overset{ˆ}{\gamma }}={\left[j^{\prime }\right]}_{\overset{ˆ}{\gamma }}$, so $j\overset{ˆ}{\gamma }j^{\prime }$ and thus $uv\overset{ˆ}{\theta }u^{\prime }v^{\prime }.\;f_{2}$ is surjective because each digit of η changes over some edge. This proves the first assertion of the theorem. For the second assertion, we use the bijection between $E(G)/\overset{ˆ}{\theta }$ and $\left\{G_{1},\ldots ,G_{n}\right\}$ that takes each $G_{i}$ to the $E_{i}\in E(G)/\overset{ˆ}{\theta }$ that was used to construct it (see the description of Algorithm 1 in Section 2). We also have a bijection $f_{3}$ from $[m]/\overset{ˆ}{\gamma }$ to the canonical partition of η, since the elements of $[m]/\overset{ˆ}{\gamma }$ were used to form that partition. Thus, the mapping $f=f_{3}\circ f_{2}\circ f_{1}$ is a bijection from the factors of the canonical isometric representation of G to the canonical partition of η.

### Proposition 3.4.

Let weighted graph G have canonical isometric representation $\pi \;:V(G)\to V\left(\prod_{i=1}^{n}\;G_{i}\right),\;\pi =\left(\pi_{1},\ldots ,\pi_{n}\right)$. Let η be a Hamming embedding of G with canonical partition $\eta^{1},\ldots ,\eta^{n}$. Assume without loss of generality that the natural bijection of Proposition 3.3 maps $G_{i}$ to $\eta^{i}$ for each i,1≤i≤n. Then for each i there is an embedding ${\tilde{\eta }}^{i}$ such that $\eta^{i}={\tilde{\eta }}^{i}\circ \pi_{i}$, which is a Hamming embedding of $G_{i}$.

### Proof.

Fix any two vertices u,v∈V(G) and consider a shortest path P from u to v. For each $G_{i}$, let $E_{i}$ be the equivalence class under $\overset{ˆ}{\theta }$ from which $G_{i}$ was generated and let $c_{i}$ be the sum of the edge weights for edges along P that are in $E_{i}$. By Lemma 3.1, for each i, there must exist in $G_{i}$ a path of length $c_{i}$, so $d_{\pi_{i}}(u,v)\leq c_{i}$. Further, each edge along P contributes to exactly one $c_{i}$, so we have

$$d_{G}\left(u,v\right)=\sum_{i=1}^{n}c_{i}=\sum_{i=1}^{n}d_{\pi_{i}}\left(u,v\right),$$

where the second equality is due to the fact that π is an isometric embedding. Thus $d_{\pi_{i}}(u,v)=c_{i}$ for each i. Now take $\eta^{i}$, for which we know, based on the construction of Proposition 3.3, that $w_{G}(e)$ digits change across any edge $e\in E_{i}$ and no digits change across any other edge. Then $d_{\eta^{i}}(u,v)\leq c_{i}$. In fact, we have

$$d_{G}\left(u,v\right)=\sum_{i=1}^{n}c_{i}=\sum_{i=1}^{n}d_{\eta^{i}}\left(u,v\right),$$

where again the second equality is because η is an isometric embedding. As before, this implies that $d_{\eta^{i}}(u,v)=c_{i}$. Thus, $d_{\eta^{i}}(u,v)=d_{\pi_{i}}(u,v)$.

We construct a Hamming embedding ${\tilde{\eta }}^{i}$ for $G_{i}$ that satisfies $\eta^{i}={\tilde{\eta }}^{i}\circ \pi_{i}.\;\pi_{i}$ is not necessarily a bijection because multiple nodes in V(G) may map to the same $u_{i}$ in $G_{i}$. However, we may let $\pi_{i}^{-1}\;:V\left(G_{i}\right)\to V(G)$ map $u_{i}\in V\left(G_{i}\right)$ to any u∈V(G) such that $\pi_{i}(u)=u_{i}$. Let ${\tilde{\eta }}^{i}=\eta^{i}\circ \pi_{i}^{-1}$. Then for $u_{i},\;v_{i}\in V\left(G_{i}\right)$ such that $\pi_{i}^{-1}\left(u_{i}\right)=u$ and $\pi_{i}^{-1}\left(v_{i}\right)=v,\;{\tilde{\eta }}^{i}\left(u_{i}\right)=\eta^{i}\left(\pi_{i}^{-1}\left(u_{i}\right)\right)=\eta^{i}(u)$, and so $d_{{\tilde{\eta }}^{i}}\left(u_{i},v_{i}\right)=d_{\eta^{i}}(u,v)=d_{\pi_{i}}(u,v)=d_{G_{i}}\left(\pi_{i}(u),\pi_{i}(v)\right)=d_{G_{i}}\left(u_{i},v_{i}\right)$. Thus, ${\tilde{\eta }}^{i}$ is a Hamming embedding of $G_{i}$.

### Theorem 3.5.

Let G be a weighted graph with $G_{1},\ldots ,G_{n}$ the factors of its canonical isometric representation. Then G is Hamming embeddable if and only if each $G_{i}$ is Hamming embeddable.

### Proof.

Let $\pi \;:V(G)\to \prod_{i=1}^{n}V\left(G_{i}\right)$ be the canonical isometric representation of G, with $\pi =\left(\pi_{1},\ldots ,\pi_{n}\right)$.

If G is Hamming embeddable then by Proposition 3.4 we may construct Hamming embeddings of each $G_{i}$.

If every $G_{i}$ is Hamming embeddable, then for each i let $H_{i}$ be a Hamming graph into which $G_{i}$ has an isometric embedding. Let $H=\prod_{i=1}^{n}H_{i}$, which is a Hamming graph because the class of Hamming graphs is closed under taking Cartesian products. As G is isometrically embeddable into $\prod_{i=1}^{n}G_{i}$, which is isometrically embeddable in H, we have that G is Hamming embeddable.

### Corollary 3.6.

A weighted graph G is hypercube embeddable if and only if each factor of its canonical isometric representation is hypercube embeddable.

### Proof.

If G has a hypercube embedding η, then each element of the canonical partition of η is also a hypercube embedding. Thus each factor of the canonical isometric representation of G has a hypercube embedding formed from an element of the canonical partition of η. Conversely, if each factor of the canonical isometric representation of G has a hypercube embedding, these may be concatenated to form a hypercube embedding of G.

The following corollary extends these results to $\ell_{1}$-embeddability. A scale-k embedding is an embedding ϕ:V(G)→V(H) in which $d_{G}(u,v)=kd_{\phi }(u,v)$. It is well-established that a rational-valued metric is $\ell_{1}$-embeddable if and only if it has a scale-k embedding into a hypercube for some k (see, e.g., [9]).

### Corollary 3.7.

A weighted graph G has a scale-k embedding into a Hamming graph if and only if each factor of its canonical isometric representation has a scale-k embedding into a Hamming graph. It follows that a graph G with rational edge weights has an $\ell_{1}$-embedding if and only if each factor is $\ell_{1}$-embeddable.

### Proof.

Assume G has canonical isometric representation $\pi \;:V(G)\to V\left(\prod_{i=1}^{n}G_{i}\right)$.

Scaling all edge weights of G by k also scales the edge weights within each factor of its canonical isometric representation by k. By Corollary 3.6, this implies the first part of the corollary.

Assume G has rational edge weights. Then each $G_{i}$ that is scale-embeddable into a hypercube must be scale-$k_{i}$ hypercube embeddable for some $k_{i}\in Z_{+}$. If $G_{i}$ has a scale-$k_{i}$ hypercube embedding for all 1≤i≤n, then let $k=\prod_{i=1}^{n}k_{i}$. Then $G_{i}$ has a scale-k hypercube embedding for each i, so G does also. The converse implication follows by applying Corollary 3.6 to a scale-k embedding of G to yield scale-k embeddings of each $G_{i}$.

### Corollary 3.8.

Given a weighted graph G, the number of non-equivalent Hamming embeddings of G is the product of the number of non-equivalent Hamming embeddings of each factor of its canonical isometric representation. Similarly, the number of non-equivalent hypercube embeddings of G is the product of the number of non-equivalent hypercube embeddings of each factor.

### Proof.

Let π be the canonical isometric representation of $G,\;\pi \;:V(G)\to V\left(\prod_{i=1}^{n}G_{i}\right)$, and $\pi =\left(\pi_{1},\ldots ,\pi_{n}\right)$.

Take any two non-equivalent Hamming embeddings η and ζ of G. Each element $\eta^{i}$ of the canonical partition of η corresponds to a Hamming embedding ${\tilde{\eta }}^{i}$ of $G_{i}$, with $\eta^{i}={\tilde{\eta }}^{i}\circ \pi_{i}$. This is similarly true of ζ. Now if, for all $1\leq i\leq n,\;{\tilde{\eta }}^{i}$ is equivalent to ${\tilde{\zeta }}^{i}$, then each ${\tilde{\eta }}^{i}$ can be made identical to ${\tilde{\zeta }}^{i}$ by permuting coordinates and coordinate values. Because $\eta^{i}={\tilde{\eta }}^{i}\circ \pi_{i}$ and $\zeta^{i}={\tilde{\zeta }}^{i}\circ \pi_{i}$, this implies that η can be made identical to ζ by the same process, so η and ζ are equivalent. Thus, non-equivalent η and ζ must have some ${\tilde{\eta }}^{i}$ not equivalent to ${\tilde{\zeta }}^{i}$. That is, any non-equivalent η and ζ will correspond to Hamming embeddings of the $G_{i}$ that are distinct under equivalence for at least one i, so the product of the number of non-equivalent Hamming embeddings of the $G_{i}$ is at least the number of non-equivalent Hamming embeddings of G.

Now let ${\tilde{\eta }}^{1},\ldots ,{\tilde{\eta }}^{n}$ and ${\tilde{\zeta }}^{1},\ldots ,{\tilde{\zeta }}^{n}$ be such that each ${\tilde{\eta }}^{i}$ and ${\tilde{\zeta }}^{i}$ are Hamming embeddings of $G_{i}$. Let $\eta^{i}={\tilde{\eta }}^{i}\circ \pi_{i}$ and $\zeta^{i}={\tilde{\zeta }}^{i}\circ \pi_{i}$, and consider the concatenations of the $\eta^{i}$ and the $\zeta^{i},\;\eta =\eta^{1}\cdots \eta^{n}$ and $\zeta =\zeta^{1}\cdots \zeta^{n}$. Note that η and ζ form Hamming embeddings of G. If η and ζ are equivalent, then η may be made identical to ζ by permuting coordinates and coordinate values. Note that any such permutation must map each coordinate in $\eta^{i}$ to a coordinate in $\zeta^{i}$, because the corresponding digits necessarily change across the same edges. This allows the permutation from η to ζ to be decomposed into permutations from $\eta^{i}$ to $\zeta^{i}$, so each ${\tilde{\eta }}^{i}$ and ${\tilde{\zeta }}^{i}$ must be equivalent. From this, we conclude that, for any 1≤i≤n, if ${\tilde{\eta }}^{i}$ and ${\tilde{\zeta }}^{i}$ are not equivalent then their corresponding η and ζ generated by the above process are also not equivalent. This indicates that the number of non-equivalent Hamming embeddings of G is not less than the product of the number of non-equivalent Hamming embeddings of the $G_{i}$. This completes the proof.

This can be proven identically for counting hypercube embeddings, noting that if η and ζ are hypercube embeddings then each element of their canonical partitions is also a hypercube embedding.

## 4. Conclusion

Weighted graphs are capable of representing a richer variety of distance relationships than unweighted graphs. Yet the added complexity of weighted graphs has made it difficult to develop a deep understanding of isometric embeddings of weighted graphs into various destination graphs of interest, such as unweighted Hamming graphs.

Here, we applied the canonical isometric representation of an arbitrary weighted graph in a Cartesian product of irreducible weighted graphs to investigate the structure of embeddings into hypercube graphs, Hamming graphs, and the metric space $\ell_{1}$. Distances in a Cartesian graph product may be decomposed additively into distances within the factor graphs, which allows an isometric embedding of the product graph to be formed from isometric embeddings of the factor graphs. For example, with approximate embeddings this permits an extension of Bourgain’s Theorem that any metric space on n points may be embedded into the metric space $\ell_{1}$ with O(logn) distortion to allow such an embedding with $max\{O\left(\log n_{i}\right)\}$ distortion, where $n_{i}$ is the number of vertices in the ith factor of the canonical isometric representation. For isometric embeddings, our results show that in fact *every* Hamming embedding (up to equivalence) must be formed as a concatenation of Hamming embeddings of each factor in its canonical isometric representation. Although a polynomial-time algorithm for deciding hypercube embeddability is unlikely to exist [7], this eases the task of finding Hamming embeddings, and of proving their non-existence, in cases where the graph has a nontrivial isometric representation. Future work may further characterize the classes of graphs for which we can decide Hamming or hypercube embeddability in polynomial time.

More generally, isometric embeddings of weighted graphs into arbitrary unweighted graph products remain relatively unstudied, and it is unknown to what extent our results here generalize to this context. For example, with Hamming embeddable graphs our results imply a hierarchical decomposition of a weight-minimal graph into a Cartesian graph product, whereby a graph may be decomposed first into a Cartesian product of weighted graphs (its canonical isometric representation) and then each factor of this product may be individually decomposed into a Cartesian product of unweighted complete graphs. A similar hierarchy for embeddings into arbitrary unweighted graph products could take the following form: For weighted graph G, let $G_{1},\ldots ,G_{n}$ be the factors of its canonical isometric representation. Then, for any isometric embedding of G into a Cartesian product of irreducible unweighted graphs $\prod_{i=1}^{m}H_{i}$, is there a partition of [m], $\left\{J_{1},\ldots ,J_{n}\right\}$, for which $G_{i}$ is isometrically embeddable into $\prod_{j\in J_{i}}H_{i}$ for each i? As with Hamming embeddings, this would imply that we can characterize all isometric embeddings of a weighted G into unweighted graphs as concatenations of isometric embeddings of the $G_{i}$ into unweighted graphs. Such a hierarchy would be an appealing and exciting result, but remains to be proven.

## Data Availability

No data was used for the research described in the article.

## Footnotes

2 This term is motivated by the fact that no edge weight may be reduced without modifying the graph’s path distance metric.

3 An isometric representation is called a *pseudofactorization* by Sheridan et al. [21], to distinguish from a factorization of a graph G, for which G is isomorphic to the Cartesian product.

4 A cocktail party graph is a graph formed from a complete graph by removing the edges in a perfect matching.

## References

[1] Antebi YE, Linton JM, Klumpe H, Bintu B, Gong M, Su C, McCardell R, Elowitz MB, Combinatorial signal perception in the BMP pathway, Cell 170 (6) (2017) 1184–1196. 10.1016/j.cell.2017.08.015 PMC5612783 28886385

[2] Avis D, Hypermetric spaces and the Hamming cone, Canad. J. Math. 33 (4) (1981) 795–802.

[3] Bandelt H-J, Retracts of hypercubes, J. Graph Theory 8 (4) (1984) 501–510.

[4] Bee C, Chen Y-J, Queen M, Ward D, Liu X, Organick L, Seelig G, Strauss K, Ceze L, Molecular-level similarity search brings computing to DNA data storage, Nature Commun. 12 (1) (2021) 4764. 10.1038/s41467-021-24991-z PMC8346626 34362913

[5] Björner A, Edelman PH, Ziegler GM, Hyperplane arrangements with a lattice of regions, Discrete Comput. Geom. 5 (3) (1990) 263–288.

[6] Chepoi V, Isometric subgraphs of Hamming graphs and d-convexity, Cybernetics 24 (1) (1988) 6–11.

[7] Chvátal V, Recognizing intersection patterns, Ann. Discrete Math. 8 (1980) 249–251.

[8] Deza M, Laurent M, ℓ_1_-Rigid graphs, J. Algebraic Combin. 3 (2) (1994) 153–175.

[9] Deza MM, Laurent M, Geometry of Cuts and Metrics, in: Algorithms and Combinatorics, vol. 15, Springer, Berlin, Heidelberg, 1997.

[10] Deza M, Shpectorov S, Recognition of the ℓ_1_-graphs with complexity O(nm), or football in a hypercube, European J. Combin. 17 (2–3) (1996) 279–289.

[11] Djoković DŽ, Distance-preserving subgraphs of hypercubes, J. Combin. Theory Ser. B 14 (3) (1973) 263–267.

[12] Eigen M, Winkler-Oswatitsch R, Transfer-RNA: the early adaptor, Naturwissenschaften 68 (5) (1981) 217–228. 10.1007/BF01047323 6909552

[13] Eppstein D, Recognizing partial cubes in quadratic time, J. Graph Algorithms Appl. 15 (2) (2011) 269–293.

[14] Feder T, Product graph representations, J.Graph Theory 16 (5) (1992) 467–488.

[15] Firsov V, Isometric embedding of a graph in a Boolean cube, Cybernetics 1 (6) (1965) 112–113.

[16] Graham RL, Pollak HO, On the addressing problem for loop switching, Bell Syst. Tech. J. 50 (8) (1971) 2495–2519.

[17] Graham RL, Winkler PM, On isometric embeddings of graphs, Trans. Amer. Math. Soc. 288 (2) (1985) 527–536.

[18] Kautz WH, Unit-distance error-checking codes, IRE Trans. Electron. Comput. EC-7 (2) (1958) 179–180.

[19] Klavžar S, Gutman I, Mohar B, Labeling of benzenoid systems which reflects the vertex-distance relations, J. Chem. Inf. Comput. Sci. 35 (3) (1995) 590–593.

[20] Laurent M, Hypercube embedding of distances with few values, in: Barcelo H, Kalai G (Eds.), Contemporary Mathematics, Vol. 178, American Mathematical Society, Providence, Rhode Island, 1994, pp. 179–207, Jerusalem Combinatorics ‘93.

[21] Sheridan K, Berleant J, Bathe M, Condon A, Vassilevska Williams V, Factorization and pseudofactorization of weighted graphs, 2021, 10.48550/arXiv.2112.06990, arXiv e-prints arXiv:2112.06990. PMC10194401 37213330

[22] Shpectorov SV, On scale embeddings of graphs into hypercubes, European J. Combin. 14 (2) (1993) 117–130.

[23] Wilkeit E, Isometric embeddings in Hamming graphs, J. Combin. Theory Ser. B 50 (2) (1990) 179–197.

[24] Winkler PM, Isometric embedding in products of complete graphs, Discrete Appl. Math. 7 (2) (1984) 221–225.

